# Harmonic Structure Predicts the Enjoyment of Uplifting Trance Music

**DOI:** 10.3389/fpsyg.2016.01999

**Published:** 2017-01-10

**Authors:** Kat Agres, Dorien Herremans, Louis Bigo, Darrell Conklin

**Affiliations:** ^1^Centre for Digital Music, Electronic Engineering and Computer Science, Queen Mary University of LondonLondon, UK; ^2^Institut de Recherche et Coordination Acoustique/MusiqueParis, France; ^3^Department of Computer Science and Artificial Intelligence, University of the Basque Country UPV/EHUSan Sebastián, Spain; ^4^IKERBASQUE, Basque Foundation for ScienceBilbao, Spain

**Keywords:** music cognition, enjoyment, repetition, complexity, Wundt curve, computational creativity, uplifting trance

## Abstract

An empirical investigation of how local harmonic structures (e.g., chord progressions) contribute to the experience and enjoyment of uplifting trance (UT) music is presented. The connection between rhythmic and percussive elements and resulting trance-like states has been highlighted by musicologists, but no research, to our knowledge, has explored whether repeated harmonic elements influence affective responses in listeners of trance music. Two alternative hypotheses are discussed, the first highlighting the direct relationship between repetition/complexity and enjoyment, and the second based on the theoretical inverted-U relationship described by the Wundt curve. We investigate the connection between harmonic structure and subjective enjoyment through interdisciplinary behavioral and computational methods: First we discuss an experiment in which listeners provided enjoyment ratings for computer-generated UT anthems with varying levels of harmonic repetition and complexity. The anthems were generated using a statistical model trained on a corpus of 100 uplifting trance anthems created for this purpose, and harmonic structure was constrained by imposing particular repetition structures (semiotic patterns defining the order of chords in the sequence) on a professional UT music production template. Second, the relationship between harmonic structure and enjoyment is further explored using two computational approaches, one based on average Information Content, and another that measures average tonal tension between chords. The results of the listening experiment indicate that harmonic repetition does in fact contribute to the enjoyment of uplifting trance music. More compelling evidence was found for the second hypothesis discussed above, however some maximally repetitive structures were also preferred. Both computational models provide evidence for a Wundt-type relationship between complexity and enjoyment. By systematically manipulating the structure of chord progressions, we have discovered specific harmonic contexts in which repetitive or complex structure contribute to the enjoyment of uplifting trance music.

## 1. Introduction

The interplay between repetition and variation is a fundamental element of music. Music cognition researchers and musicologists alike have long cited the importance of patterns, implications, and expectations, as well as violations of all of these, in creating emotional responses during music listening (Meyer, 1957; Narmour, 1990; Huron, 2006). As musical elements are repeated or varied, there is a corresponding impact on the internal prediction mechanisms responsible for the expectations (and surprise) thought to underlie affective responses in listeners (Huron, 2006). Although researchers have used a diverse set of methods to capture how elements of musical structure impact emotion, many approaches focus on quantifying the complexity of the music.

As discussed by Gaver and Mandler (1987), “the levels of structure involved [in the music] and the degree of change within and between levels, determine the complexity of the piece. This complexity can be described in terms of the information content of the music, using the term ‘information’ as it is used in information theory.” Indeed, the use of information theory to quantify the complexity of musical stimuli has been used for decades (see for example, Vitz, 1966; Werbik, 1971; Abdallah and Plumbley, 2009). Gaver and Mandler (1987) refer to information as redundancy, stating that, “If the next note in a piece of music is relatively determined by that which has gone before, it conveys little new information about the piece. A work with complicated changes… can be said to contain more information than a piece that is relatively repetitive…” To examine how the structure of music impacts listeners' affective responses, researchers have assessed how repetitive or complex particular aspects of the music are, and test musical stimuli that vary from repetitive, on one end of the spectrum, to complex, on the other end of the spectrum.

### 1.1. Repetition, complexity, and the wundt curve

Previous studies have shown that humans typically display a subjective preference for a certain amount of variation or complexity in auditory or visual stimuli (described by Heyduk, 1975 as “optimum complexity”). Stimuli that are less or more complex than the individual's subjective optimum are less preferred, thus forming a bell-shaped preference distribution known in the literature as the Wundt curve. As described by Berlyne (1970), this inverted-U curve indicates that a novel and complex stimulus is likely to be judged as unpleasant, and “repetition should make it progressively less unpleasant and finally more and more pleasant until, after reaching a peak of pleasantness, it should become indifferent.” The inverted-U relationship between pleasure (or liking or enjoyment) and complexity has been demonstrated for several musical genres, and with differing approaches to the experimental manipulation of complexity or repetition of stimuli (Vitz, 1966; Heyduk, 1975; North and Hargreaves, 1995; Huron, 2006; Temperley, 2014; Witek et al., 2014). For example, Moles (1966) applied this principle to information theory, using information theoretic measures to define complexity on the Wundt curve, suggesting that too little complexity produces boredom, while too much complexity is difficult and unpleasant to process. Similarly, Simonton (2001) observed an inverted-U relationship between melodic originality and enjoyment such that maximal musical enjoyment is elicited when melodies occur in the middle of the range of melodic originality. In the current research, we consider the Wundt curve to reflect a range of enjoyment (rather than associated constructs such as boredom), and we elicit enjoyment ratings from participants.

In addition to using information content to measure the complexity of a piece, harmonic complexity may also be defined in terms of movement between chords in tonal space. Using computational modeling, this can be captured as the Euclidean distance between the geometrical centers of two subsequent chords in tonal space (Chew, 2013), as described in the spiral array model of tonal tension (Herremans and Chew, 2016a). The concept of tension has been studied extensively in the context of expectations (Lerdahl, 2004; Margulis, 2005; Huron, 2006; Farbood, 2012), and we hypothesized that a similar inverted U-shaped curve describes the relationship between tonal tension and enjoyment.

### 1.2. Repetition and trance music

In contrast to the inverted-U relationship between complexity and enjoyment discussed above, some musical genres appear to limit variation and complexity, with the aim of producing aesthetic responses (and even changes in phenomenological states) based on long stretches of highly repetitive music. For example, the repetition inherent in various forms of trance music is thought to be crucial for evoking alternative listening states (see Walsh, 1989), such as heightened enjoyment, prolonged periods of pleasure, and a sense of becoming “lost in the music” (Garcia, 2005; Sacks, 2006). Some neurophysiological evidence supports these phenomenological reports of trance music listening: Intense activation or repetitive hyper-stimulation of the temporal lobe, hippocampus, and amygdala can induce altered states of consciousness (Joseph, 1992), and repetitive rhythmic elements can prompt brain networks to be dynamically reconfigured, potentially underlying trance states (Hove et al., 2016). That is, repetition seems to play a particularly important role in underlying the phenomenological experiences that likely contribute to listeners' motivation for listening to this genre, and that drive enjoyment of trance music. This begs the question of whether all aspects of trance music are most effective (for reaching heightened enjoyment) when they are very repetitive, or whether some musical elements still adhere to the balance between repetition and variation described by Wundt and Berlyne.

Whereas previous research has focused upon the intuitive connection between repeated rhythmic/percussive elements and the physiological entrainment underpinning heightened enjoyment and trance states (Neher, 1962; Becker-Blease, 2004; Fachner, 2011; Becker, 2012; Trost et al., 2014; Hove et al., 2016), the particular repeated elements influencing affective response in listeners remain unclear, and, to our knowledge, no research has hitherto explored the relative influence of harmonic repetition on affective or physiological responses to trance music. The present study would provide the first evidence, to the authors' knowledge, that harmonic structure contributes to the enjoyment of trance music.

To address the question of harmonic structure on enjoyment, we focused on a sub-genre of electronic dance music called Uplifting Trance (UT), which is characterized by the use of repetitive tones and chord sequences within repeated rhythmic patterns (Madrid, 2008). UT pieces include sections such as the breakdown, the buildup, and the anthem, and each of these parts is intended to have a particular function or impact on audience perception and affective responses. In this study, we focused on one of the most distinctive and fundamental elements of a UT piece: the anthem, arguably the energetic height of the UT listening experience. The anthem in a UT piece can be compared to the chorus in a popular song. It generally features the majority of instruments appearing in the piece, and is intended to be catchy and memorable. UT anthems are typically preceded by two consecutive sections called the “breakdown” and “buildup,” respectively. In the former, the energy is gradually decreased, after which there is a build up of energy that culminates in a powerful anthem. For a more detailed description of the functional sections of trance music, the reader is referred to Conklin (2016). By generating excerpts of UT anthems varying in repetitiveness and harmonic complexity, we were able to investigate whether repetitive harmonic patterns (i.e., chord progressions) influence the enjoyment of trance music such that more repetitive structures lead to greater enjoyment, or harmonic repetition in this genre has a Wundt-type relationship to listeners' enjoyment akin to other more varied genres such as classical and jazz. Although we were limited to using only brief excerpts (to allow time to present a range of stimuli), and caution is therefore needed in generalizing the results to all of trance music listening, the sequence of chords found in the anthem section is often identical to those found in the other main sections of a trance piece (i.e., the breakdown and the buildup), providing some ground for generalization.

## 2. Behavioral experiment: repetitive structures and enjoyment

To elucidate the connection between repetition of harmonic structure and subjective enjoyment, we conducted a behavioral experiment in which listeners provided enjoyment ratings for UT anthems varying in harmonic repetition (specifically, the repetitiveness of chord sequences). The chord sequences generated for this experiment varied in terms of pre-defined semiotic structures (e.g., patterns of chords), which allowed us to examine how types of complexity or repetition impact enjoyment of the sequences. In this work, semiotic patterns are represented as *patterns with variables* (Angluin, 1980; Conklin, 2016), where occurrences of the same variable in a pattern must be substituted by the same chord. For example, if a semiotic pattern has the structure ABCD-BCDB, the chord substituted for ‘B’ must be the same for all three instances of that variable in this pattern (note that a different chord may be substituted for ‘B’ in a different semiotic pattern). Furthermore, no chord is permitted to substitute for more than one variable in a pattern.

**Figure 1:**
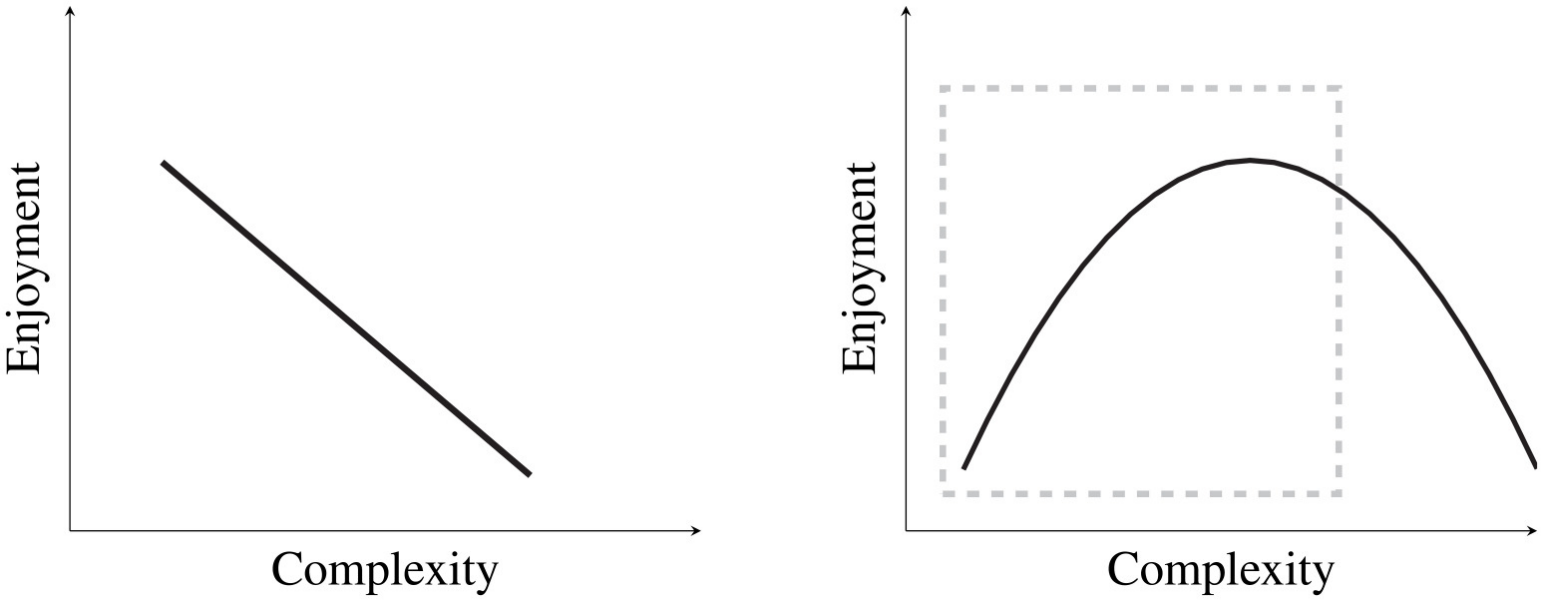
**Depicted on the left is Hypothesis 1, that enjoyment decreases as stimuli become less repetitive and more complex**. Depicted on the right is Hypothesis 2, which displays the relationship between preference and complexity as predicted by the Wundt curve. Because no very complex stimuli were included in this research, the study only addresses the portion of the curve outlined by the dashed gray box.

We approached this work with two alternative hypotheses: First, that listeners display a preference for repetitive harmonic structures such that more varied/complex patterns yield less enjoyment, as shown in the left plot of Figure 1, and second, that enjoyment reflects the inverse-U relationship between preference and complexity predicted by the Wundt curve (Berlyne, 1970) (shown in the right plot of Figure 1).

Very complex or unpredictable sequences (that should yield very low enjoyment ratings according to the right-hand portion of the Wundt curve) were not included in this study, because the research aimed to maintain as much ecological validity as possible, and because generating very complex sequences was often not possible given the simple semiotic structures employed (see next section for details). As a result, this research addresses a range of stimuli from extremely repetitive to fairly complex, approximately the portion of the Wundt hypothesis outlined by the dashed gray box in Figure 1. In essence, the prediction here is that stimuli with less repetitiveness or greater harmonic complexity will generally result in greater enjoyment, although the *most* complex stimuli should not result in the highest enjoyment ratings (the most complex stimuli correspond to the right of the apex of the Wundt curve, or the righthand side of the dashed box in Figure 1).

We employed three approaches to quantifying the harmonic structure of stimuli. First, we categorized the harmonic repetitiveness of stimuli using three categorical repetition variables (specific to our stimulus set). Each of these three provided a value ranging from “very repetitive” to “not repetitive,” and stimuli that were not repetitive were considered to be more complex. Second, we used Information Content to measure the average unpredictability and complexity of each sequence (see Sections 2.1.4.2 and 3.1). And third, we computed complexity as the average tonal tension measured between subsequent chords of each stimulus (described in Section 3.2).

To test the two hypotheses above, we implemented two strategies. As discussed in the present section, we first examined the relationship between participants' enjoyment ratings and the three *categorical* repetition variables mentioned above (and discussed in detail below). Then, as described in Section 3, we used computational approaches to examine the relationship between participants' ratings and two *continuous* measures of complexity: *average information content* and average tonal tension (or *Cloud Momentum*, discussed in depth in Section 3). Note that testing continuous variables allowed us to more easily compare the fit of both linear and quadratic functions (corresponding to hypotheses 1 and 2) in accounting for variance in the participants' responses.

### 2.1. Methods

#### 2.1.1. Participants

Email advertisements and flyers around the Queen Mary University of London campus were used to recruit twenty volunteers for the experiment (mean age = 28.6 years, std = 7.9 years; 6 female and 14 male). The resulting group of participants consisted of undergraduate and graduate students. Each of these reported prior experience listening to trance music, and compensation of £7 was given for participation in the experiment. Participants with experience listening to trance music were chosen to mitigate the effect of familiarity on enjoyment, as described by Steck and Machotka (1975).

#### 2.1.2. Stimuli

In order to vary the repetition/complexity of stimuli in the experiment, chord generation for every UT excerpt was constrained by an underlying semiotic pattern which governed the repetition structure of the music. The 14 different semiotic patterns are used in this study (as described below). Please note that the labels A, B, C, etc., indicate the order and repetition of chords, and do not refer to explicit chord names. Every stimulus has a duration of 16 bars and is comprised of 8 chords (each chord has a duration of two bars).

In order to create stimuli with adequate variation in semiotic structure, several guidelines were followed when creating the semiotic patterns. These guidelines addressed the degree of repetitiveness in the first half of the stimulus, the degree of repetitiveness in the second half of the stimulus, and finally, the amount of similarity between both halves of the stimulus (repetitiveness in musical form). Note that higher-level musical repetition (such as an 8-bar pattern that is repeated several times) was not explored in this research. Note also that a full-factorial design was not possible, given the repetition features themselves (see below), and in order to limit listener fatigue (the stimuli were each 30 s in duration, and only so many semiotic structures could be tested). Accordingly, the semiotic patterns were constrained by the following features:

The first half (first four chords) of the semiotic pattern was either of the form ABCD or AABB, with the later structure considered more repetitive and less varied than the former. In order to maintain structure from the first half of the stimulus in the second half (to better mirror actual pieces found in the trance corpus), sequences beginning with AABB had to have at least two repeated chords in the second half of the stimulus, and sequences beginning ABCD did not have any successive repeated chords in the second half. Given this stipulation, the number of unique (i.e., non-repeated) chords in the second half of the semiotic structure ranged from 1 chord (AAAA) to 4 chords (ABCD or EFGH), with 1 unique chord representing maximal repetitiveness, and 4 chords representing maximal chord diversity and complexity. Note that semiotic patterns beginning with AABB could therefore not have 4 unique chords in the second half, and semiotic patterns beginning with ABCD could therefore not have 1 unique chord in the second half. Then, when comparing both halves of the semiotic structure, there are three levels of repetition in form, when considering the particular chord *and* its metrical position in half of the semiotic pattern, such that (1) the semiotic structure of the first half is completely preserved in the second half (as in AABB-AABB and ABCD-ABCD), (2) only some of the semiotic structure of the first half is preserved (this may range from 1-2 chords, as in AABB-AACC, and in ABCD-CECF, where the repeated chords are in bold font), or (3) none of the semiotic structure of the first half is preserved (e.g., AABB-CCDE and ABCD-EFGH). Finally, in an effort to maintain ecological validity with respect to the UT corpus created for this study (as described in Section 2.1.4.1), stimuli were made to reflect native structures (i.e., semiotic patterns occurring in the corpus described in Section 2.1.4.1) wherever possible. The resulting semiotic structures used in the listening experiment are shown in Table 1.

**Table 1:** **The 14 semiotic structures used in the experiment**.

AABB-AAAA	ABCD-ABCD
AABB-AABB	ABCD-AECF
AABB-AACC	ABCD-BCDB
AABB-BBAA	ABCD-CECF
AABB-CCAA	ABCD-DABD
AABB-CCAB	ABCD-EFAB
AABB-CCDE	ABCD-EFGH

To avoid any potential confound of the actual chords presented (as opposed to the overarching semiotic structure), four different stimuli were generated for each semiotic structure listed in Table 1. Two major keys common in UT music were selected, D and G, as well as their relative minor keys, B minor and E minor. The first chord of each sequence was therefore either D major, B minor, G major, or E minor. The generation was performed using a method that allows sampling high probability chord sequences with respect to a given semiotic structure (Conklin, 2016) (see Section 2.1.4.2). To generate the chord sequences, a statistical model was used to encode chord transition probabilities from a corpus of 100 uplifting trance chord loops compiled specifically for this study (described in Section 2.1.4.1)^1^. The sequences (generated by the statistical model) with the highest probability between successive chords were selected, and key modulations within the sequences were avoided as much as possible. To create the audio files used in the listening study, the selected chord sequences were then rendered within the Digital Audio Workstation Logic Pro X (LPX) by starting from an existing uplifting trance template^2^ and applying as few as possible pitch modifications necessary to make its harmonic structure fit with the generated chord sequence (Bigo and Conklin, 2015). As this transformation task leaves rhythm, instrumentation, and audio effects unchanged, the generation results in a set of stimuli that differ primarily by their harmonic properties. The entire generation process was automated by using a system that interacts with LPX through a MIDI protocol (Conklin and Bigo, 2015).

#### 2.1.3. Procedure

During the behavioral experiment a total of 56 UT excerpts (4 instances per semiotic pattern, as described above in the previous subsection), each 30 s in duration, were presented to the participants. Every participant received a different randomized order of the stimuli. The experiment started with two practice trials in order to help familiarize the listener with the experimental procedure. After listening to each stimulus, the listener's task was to provide an enjoyment rating on a 7-point Likert scale, where 1 was equivalent to “Did not enjoy at all” and 7 represented “Enjoyed very much.”

Participants were seated in a quiet room and wore headphones, set to a comfortable volume level, to listen to the stimuli. A MacBook Pro Laptop was used for data collection, on which a GUI was installed, which was created especially for this experiment^3^. Upon completing the experimental trials, participants completed a brief demographics questionnaire and were debriefed regarding the aims of the study.

#### 2.1.4. UT stimulus generation and rendering

##### 2.1.4.1. UT trance corpus

To generate chord sequences for the UT trance anthems, a small corpus of 100 UT anthem chord loops was manually transcribed by listening to trance mixes and identifying the chord sequence loop occurring in the anthem section of each song. The artist and title of every piece from which a UT anthem was extracted are listed in Appendix A (Supplementary Material). To select the 100 pieces, a priority was given to pieces judged by the authors to be the most representative of the Uplifting Trance style. Pieces with an ambiguous harmonic interpretation were avoided.

A loop is represented in the corpus by a sequence of chords, each associated with a duration in bars, where a bar corresponds to 4 successive kicks. One instance of a loop is encoded per song. Table 2 shows an example of a corpus entry. This entry indicates the chord sequence that is repeated during the anthem section of the song *Velocity in French* by the artist *Adam Ellis*. Chord symbols are represented in the corpus by a diatonic spelled root note {C, C♯, Db…B}, an additional bass note in case of inverted chords and the chord type being systematically reduced to minor or major. The corpus includes a total of 568 chords (39% minor and 61% major), and 96% of the anthems are considered “tonally consistent,” in that all of the constituent chords may be considered to be in one key (diatonic or minor harmonic). Note that since chords are associated with a duration in bars, there are no self-self chord transitions in the corpus.

**Table 2:** **Example of a UT trance corpus entry**.

Velocity in French
Adam Ellis
Dm:1
F:1
Am:2
Dm:1
F:1
Am:1
G:1

##### 2.1.4.2. Statistical model for chord sequence generation

The method for generating stimuli is based on the work of Conklin (2016), with extensions here to accommodate loops or cyclic chord sequences. A first-order statistical model for triads was trained from the corpus (Section 2.1.4.1) using the method of *viewpoints* (Conklin and Witten, 1995; Conklin, 2010, 2016), and this model was used to generate the exact chords to be used for the sequences in the study. During the training phase, sequences in the corpus were augmented again by their first chord, to simulate a loop effect. After the statistical model generated each particular chord progression (within the constraints of the imposed semiotic pattern), the chords were then rendered as an audio file, as described below in Section 2.1.4.3.

Viewpoints are functions that compute abstract features for musical events in sequences. The viewpoint used here for chord sequence modeling was a linked viewpoint of root diatonic interval and mode shift (see Conklin, 2016 for details): for example, the chord transition from C major to D minor has a root movement of a major second (indicated as M2), and a mode change from major (M) to minor (m), hence the linked viewpoint value 〈M2, Mm〉. This viewpoint has the desired property of being invariant to diatonic root transpositions, necessary especially when creating a statistical model from a small corpus. For a specified length ℓ, for every possible chord sequence *e* = *e*_1_, …, *e*_ℓ_ the first-order statistical model provides a probability $P\left(e\right)\overset{\ell }{\underset{i=2}{\sum }}P\left(e_{i}|e_{i-1}\right)$. The *information content* of a sequence *e* is *I*(*e*) = −log_2_ *P*(*e*).

Chord sequences that instantiate a particular semiotic pattern were generated using a modified random walk method (Conklin, 2003) to minimize information content, which at each step filters the possible chords for consistency with the variable substitution assigned to previous chords.

The goal during chord sequence generation was to generate sequences that could be looped, where the first chord has a dependency on the last chord. Since the number of chords in the various semiotic patterns are variable, for a chord sequence *e* = *e*_1_, …, *e*_ℓ_, we compute the *average* (i.e., per-chord) information content in the infinite limit of repetitions of *e*, according to the following derivation:

(1) $$\begin{array}{l} \begin{array}{c} \underset{n\to \infty }{\lim}\;\frac{I\left(e^{n}\right)}{\ell \times n}=\underset{n\to \infty }{\lim}\;\frac{nI\left(e\right)+\left(n-1\right)I\left(e_{\ell }+e_{1}\right)}{\ell \times n} \end{array}\\ \;=\;\frac{I\left(e\right)}{\ell }+\frac{I\left(e_{\ell }+e_{1}\right)}{\ell }\\ \;=\;\frac{I\left(e+e_{1}\right)}{\ell } \end{array}$$

To generate chord sequences for rendering, for each pattern of Table 1, four chord sequences were generated: two starting with a major triad, and two with a minor triad. The four sequences were generated using 1000 iterations of random walk. The two sequences with the lowest cyclic information content were selected from each semiotic pattern (and key). In instances where the two sequences were almost identical to one another, the next-lowest information content chord sequence was selected instead.

##### 2.1.4.3. Template and rendering

A professional Uplifting Trance piece from an Electronic Dance Music (EDM) composer was used as a *template* to produce the different generated chord sequences within the context of realistic UT anthem audio sequences. The availability of the native piece^4^ as an editable file within the Digital Audio Workstation (DAW) Logic Pro X (LPX) made possible the modification of the pitch of its notes, resulting thus in the transformation of its native chord sequence. DAW files typically consist of a set of parallel tracks (either audio or MIDI) along a common time line. Each track is attributed to a particular instrument (e.g., drums, bass, synth, etc.) and can contain digital sound effects. Audio tracks were conserved only if they did not contribute directly to the native chords, and removed otherwise. Midi tracks were all conserved.

**Figure 2:**
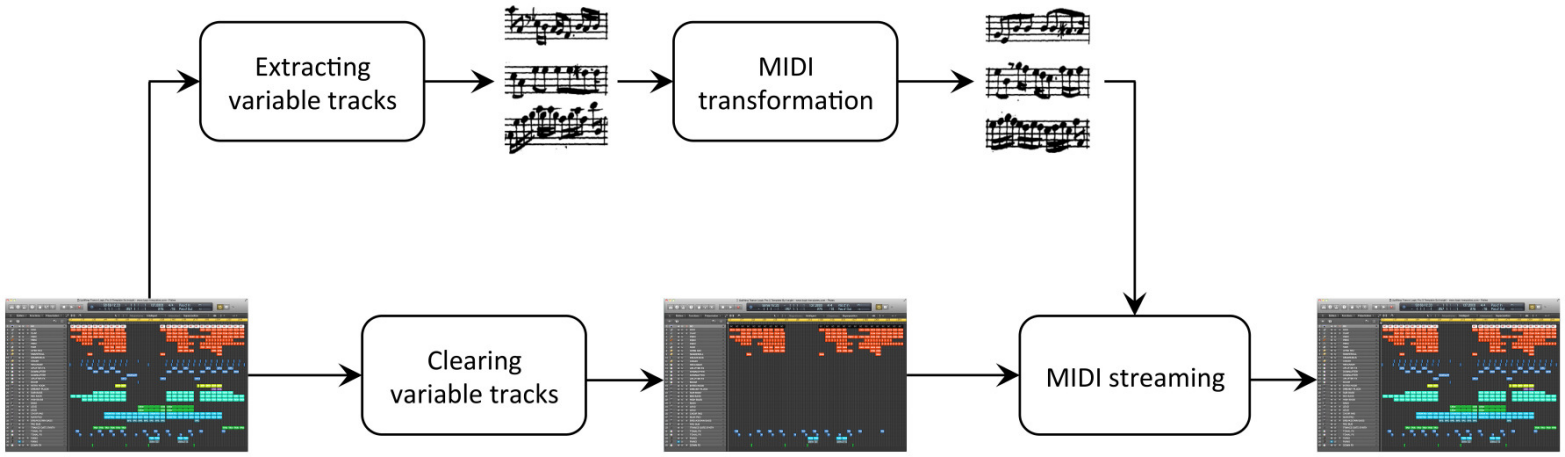
**Transformation of a LPX template file**. The original MIDI tracks are extracted from the template, transformed, and then imported into their original position in the template.

In order to adapt the template file according to a given chord sequence (of a particular semiotic pattern), the individual notes of the native chords (which are formed by arpeggiated patterns and complex melodic lines) must be modified in the MIDI tracks of the LPX template. This task was realized by: (1) exporting the original template MIDI track; (2) updating the pitch of each note in the chord to match the new chord, given its position in the original chord, and (3) re-integrating the transformed MIDI tracks in the LPX file. Task 1 can be directly performed through the “export” function of LPX. Task 2 was performed with an algorithm inspired by a dedicated harmonic transformation method developed by Conklin and Bigo (2015). Finally, for task 3, a dedicated java library was developed to automate the process of streaming midi tracks into LPX files. The chord transformation process is illustrated in Figure 2.

We now expand upon task 2. Substituting a chord by another in a MIDI file requires the pitch of each note that occurs when a chord sounds to be updated. These pitch modifications were constrained so as to keep the pitch of the notes within their original pitch range. The harmonic function of each note (i.e., whether the pitch corresponds to the root, the third or the fifth of the chord) was also conserved during the pitch transformation. For instance, the F of a D minor chord was transformed into an E if the substituting chord was a C major, so that its harmonic function (i.e., the third of the chord) would be conserved. This method also ensured that chord inversions occurred in the same places as they did in the template piece. Prior to the transformation, the template MIDI tracks were simplified such that non-chord notes, although very rare in the template anthem, were moved to the closest tone from the current chord. As a consequence, every pitch could be transformed without ambiguity.

Applying this process to each MIDI track of the native LPX file enabled each transformed chord sequence to conserve the native production features of the original track, such as digital instruments, audio effects, etc. This system was used to render each of the 56 generated UT anthems (and the two practice stimuli) as a real UT audio file.

### 2.2. Results

To test the impact of repetition on enjoyment ratings, a 2 × 4 × 3 ANOVA was conducted for three types of repetition constraining the semiotic structures described above. These three repetition variables are: (1) First Half Semiotic Structure: the structure of the first half of the semiotic pattern (AABB or ABCD), (2) Second Half Repetition: the number of unique chords in the second half of the semiotic pattern (ranging from 1 to 4 chords), and (3) Structural Overlap: the number of chords in common, *and* in the same metrical position, between the first and second halves of the sequence. This last variable was discretized into three levels, including “All Same” (4 chords in common), “Partially Same” (1 or 2 chords in common), or “All Different” (0 chords in common).

**Figure 3:**
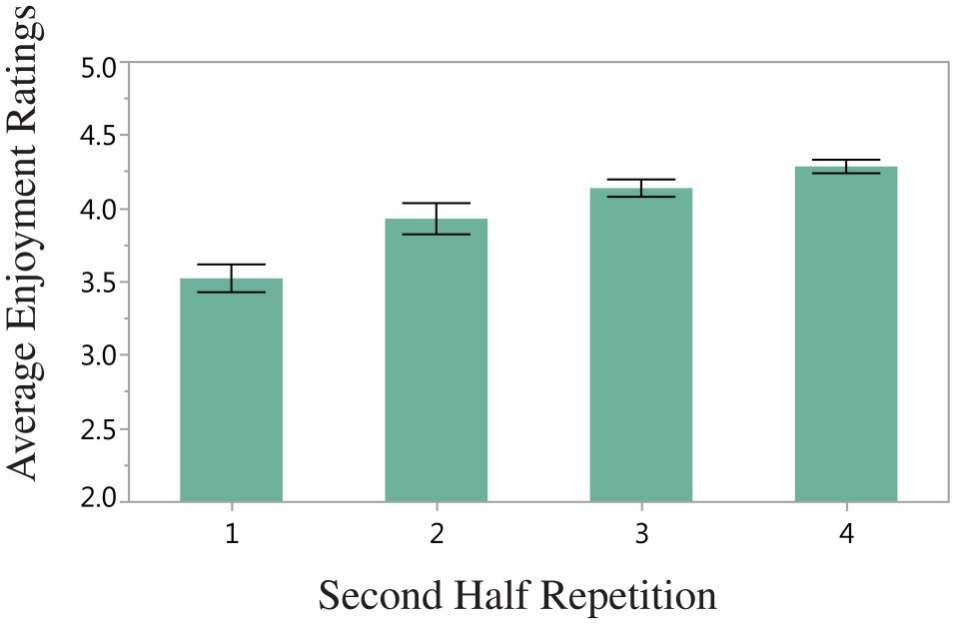
**Average enjoyment ratings for Second Half Repetition (the number of unique chords in the second half of the stimulus)**. Error bars display standard error of the mean.

**Figure 4:**
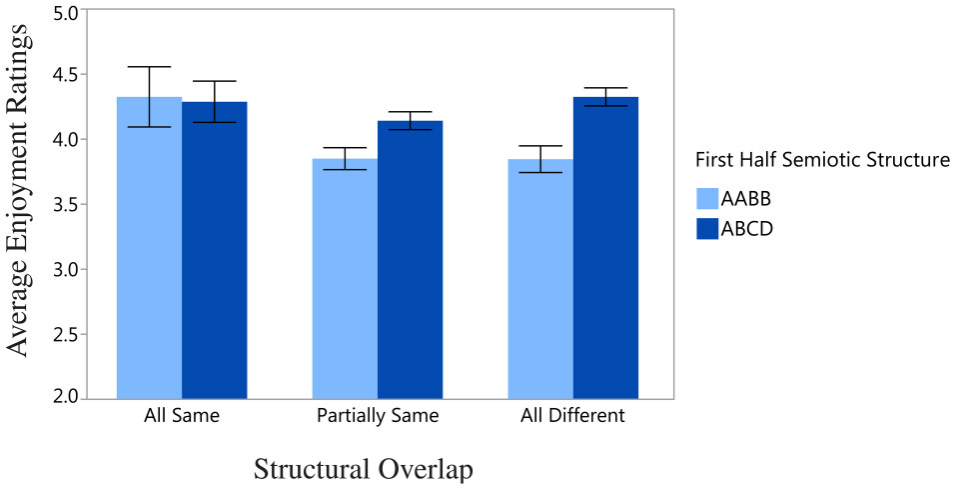
**Significant interaction between First Half Semiotic Structure (the semiotic structure of first half of the stimulus) and Structural Overlap (chords in common between the first and second halves of the stimulus)**. Error bars display standard error of the mean.

There was a significant effect of Second Half Repetition, *F*_(3, 47)_ = 4.24, *p* < 0.01, such that greater chord diversity yields higher enjoyment ratings (illustrated in Figure 3)^5^. Although First Half Semiotic Structure and Structural Overlap did not yield significant main effects, a significant interaction was found between these two variables, *F*_(2, 47)_ = 4.09, *p* < 0.05, which is depicted in Figure 4. Here, the highest enjoyment ratings are elicited when all of the same chords are present (and in the same positions) in the first and second halves of the stimulus (i.e., AABB-AABB and ABCD-ABCD), and when none of the same chords are present in the first and second half of the stimulus (for stimuli beginning with ABCD only). The lowest enjoyment ratings are elicited when the stimulus begins with AABB and the second half of the stimulus contains only partially the same or none of the same chords as the first half. These results are discussed further in the Discussion. There was no significant effect of either Mode, *F*_(1, 46)_ = 0.94, *p* = *n*.*s*., or Key, *F*_(3, 46)_ = 0.63, *p* = *n*.*s*., when these factors were added to the ANOVA analysis.

Finally, Table 3 displays the semiotic structures listed from highest to lowest average enjoyment rating.

**Table 3:** **Semiotic structures ranked from highest average enjoyment rating to lowest (standard deviations provided in parentheses)**.

**Semiotic structure**	**Average enjoyment rating**
ABCD-BCDB	4.38 (0.35)
AABB-AABB	4.33 (0.40)
ABCD-EFAB	4.33 (0.13)
ABCD-ABCD	4.29 (0.27)
ABCD-EFGH	4.28 (0.10)
ABCD-AECF	4.26 (0.14)
ABCD-DABD	4.09 (0.17)
AABB-CCAB	4.09 (0.14)
ABCD-CECF	4.08 (0.29)
AABB-CCDE	4.08 (0.06)
AABB-CCAA	4.01 (0.21)
AABB-AACC	3.94 (0.13)
AABB-AAAA	3.53 (0.17)
AABB-BBAA	3.45 (0.25)

*The horizontal line indicates the mean Enjoyment rating, e.g., semiotic structures eliciting higher than average Enjoyment ratings are above the line*.

### 2.3. Discussion

Several interesting findings emerge from the results of the three discrete repetition variables. The significant effect of Second Half Repetition indicates an overall trend of increasing enjoyment for stimuli with less repetition (i.e., more unique chords) in the second half of the stimulus. One must keep in mind, however, that only stimuli beginning AABB could have 1 unique chord in the second half, and only stimuli beginning ABCD could have 4 unique chords in the second half; therefore, the type of semiotic structure of the first half of the stimulus may be contributing to this result. The finding can therefore be explained in part by the very low Enjoyment ratings elicited by stimuli of the form AABB-AAAA (the second lowest-rated semiotic structure in the study). This may be due to a perceived violation of the implied stylistic form, e.g., culminating a semiotic pattern with AA when a change of chords is expected in the third chord position of the second half of the stimulus. The violation of implied stylistic form may also account for the relatively low ratings of stimuli with two unique chords in the second half, because sequences that begin with AABB but culminate with BBAA violate the form implied by the first half of the stimulus (in this case, the listener would expect a chord change at the first position of the second half of the stimulus, but instead hears a repetition of BB). Note that in contrast to AABB-BBAA stimuli (rated the *least* enjoyed semiotic structures of the study), stimuli of the semiotic form AABB-AABB were the second *most* enjoyed stimuli of the study, despite having similar features (including two unique chords in the second half). Again, the semiotic structure of the first half of the stimulus may have contributed to this result. Further, it is conceivable that the total number of chord changes (of which there is three in AABB-AABB and only two in AABB-BBAA) influences enjoyment, however this measure would also be contingent on the structure of the first half of the stimulus. A useful direction for future research would be to carefully construct stimuli to specifically test how the number of chord changes influences enjoyment of trance sequences. Therefore, although the result of Second Half Repetition lends some support for a preference for more varied and complex structures, future research is needed to clarify the influence of stylistic form on affective response, and to disentangle this aspect from repetition structure more generally.

The interaction between First Half Semiotic Structure (the semiotic structure of first half of the stimulus) and Structural Overlap (chords in common between the first and second halves of the stimulus) provides further evidence that the structure of the first half of the stimulus sets up expectations and preferences for the second half of the stimulus *that impact enjoyment*. Specifically, the results indicate that the greatest enjoyment ratings are elicited by semiotic patterns that are either very repetitive, namely, AABB-AABB and ABCD-ABCD, or fairly complex, such as ABCD-EFAB and ABCD-BCDB. Sequences beginning AABB are greatly enjoyed when the second half is identical to the first half, but when the second half is partially or completely altered (as in AABB-AACC, AABB-CCDE, AABB-AAAA, etc), listeners assign low enjoyment ratings to the stimuli.

Overall, the results based on categorical repetition variables provide limited support for both of the two hypotheses; therefore, we speculate that listeners enjoyment reflects a combination of the two. Although evidence was not found across all semiotic patterns to support the first hypothesis (that greater repetition yields greater enjoyment), some of the most enjoyed sequences in the study were those with complete repetition between stimulus halves (AABB-AABB and ABCD-ABCD). The second hypothesis, that enjoyment increases as complexity increases, up to a certain point (the climax of the Wundt curve), after which enjoyment decreases as complexity increases, was also supported in part by the following findings: (1) stimuli containing the least repetition in terms of diversity of chords (AABB-AAAA) were not enjoyed, (2) increasing chord diversity in the second half of the stimulus led to higher enjoyment ratings (reflecting the upward slope of the lefthand portion of the Wundt curve), (3) the semiotic structures beginning with greater complexity (ABCD) were enjoyed more than those with greater repetition (AABB), and (4) the most complex (least repetitive) semiotic pattern according to the three repetition variables, ABCD-EFGH, was not rated with the highest enjoyment (rather, it was rated fifth overall in enjoyment out of the 14 semiotic patterns), suggesting that this pattern was already on the downward slope after the apex of the Wundt curve.

In sum, these results seem to provide greater support for our second hypothesis, that the relationship between repetitive harmonic structures and enjoyment may be described by the Wundt curve. To clarify this somewhat complex set of results for the categorical repetition variables, we use continuous measures of complexity in the subsequent computational modeling section. These measures capture aspects of repetition/complexity not encoded in the discrete, hard-coded repetition variables, and have the advantage of allowing us to directly test whether the relationship between complexity and Average Enjoyment Ratings is linear or U-shaped.

## 3. Computational modeling of behavioral results: information content and tension as measures of complexity

We employ two computational approaches to model how variation in harmonic structure may account for enjoyment in listeners. The first approach uses *Information Content* (as defined in Section 2.1.4.2) as a measure of predictability and complexity. The second computes *Cloud Momentum*, derived from the spiral array model of tonal tension (Herremans and Chew, 2016a) as a measure of complexity in terms of the amount of movement in tonal space. Tension is a composite concept that encompasses many aspects of music, including rhythm, loudness, tonality, and timbre (Farbood, 2006). Because most of these aspects of tension are kept invariate in this study, we chose to employ a model that captures the tonal component of tension^6^. The tension/relaxation aspect of music is typically caused by the violation or completion of expectation when listening to music (Lerdahl, 2004; Livingstone et al., 2005; Margulis, 2005).

Although both tension and Information Content are related to the perceived expectedness of the musical signal (Margulis, 2005), these measures were selected because they capture different aspects of complexity in music (which are therefore computed differently), and because both information content and cognitive tension models have been shown to account for affective responses in listeners (Huron, 2006; Abdallah and Plumbley, 2009).

Information-theoretic measures have been used to explore affective responses to music (Meyer, 1957; Gaver and Mandler, 1987) and, more specifically, the inverse-U relationship between the predictability or variability of a signal (using measures such as entropy or information content) and aesthetic value or pleasantness (Vitz, 1966; Abdallah and Plumbley, 2009). Information content in particular has been used in several studies as a measure of unpredictability, or perceptual surprise (Vitz, 1966; Abdallah and Plumbley, 2009; Pearce and Wiggins, 2012). The more complex and unpredictable the sequence, the higher the information content.

The aspect of tension captured by *Cloud Momentum* reflects the movement in tonal space, as defined by the spiral array three-dimensional model for tonality (Chew, 2013). It is related to the feature ‘distance between chords’ as defined in the model by Lerdahl and Krumhansl (2007). We have included it in this study to capture complexity in terms of tension through movement in tonality between the chords within each stimulus (rather than the average complexity of the sequence as measured by information content). The research of Huron (2006) confirms the relationship between tension and emotions. Krumhansl (2002) also observed that increasing tension is strongly correlated with three basic emotions (happiness, sadness and fear). However, Livingstone et al. (2005) points to the fact that “too much tension can be a bad thing.” In the current modeling of tonal tension, we predict an inverse-U relationship between tension and enjoyment, whereby the optimal value in the required tension for obtaining maximal enjoyment is somewhere in the middle of the range.

Because these measures are continuous, we are able to formally test whether complexity has a negative correlation or inverted-U relationship with participants' enjoyment ratings. This will give us insight into how stimuli that fall in different places on the repetitiveness-complexity spectrum tend to be enjoyed by listeners.

### 3.1. Information content model

The model employed here to quantify *information content* is the same as that described above in Section 2.1.4.2. The average information content of a sequence is computed as presented in Equation (1).

#### 3.1.1. Information content results

The results of the linear and quadratic regression analyses, corresponding to hypotheses 1 and 2, respectively, are reported below.

The linear regression indicates that *Average Information Content* explains a significant proportion of the variance in Average Enjoyment Ratings [$R^{2}=0.39,\;F_{\left(1,\;54\right)}=34.77,\;p<0.001$]. In this analysis, *Average Information Content* significantly predicts Enjoyment Ratings [β = 0.62, *t*_(54)_ = 5.90, *p* < 0.001], however, the *R*^2^ is lower than that of the quadratic regression reported below.

The results of the quadratic regression indicate that *Average Information Content* explains a significant proportion of the variance in Average Enjoyment Ratings [$R^{2}=0.44,F_{\left(2,\;53\right)}=20.62,\;p<0.001$], where *Average Information Content* significantly predicts Enjoyment Ratings [β = 0.57, *t*_(53)_ = 5.31, *p* < 0.001], as does (*Average Information Content*)^2^ [β = −0.22, *t*_(53)_ = −2.08, *p* < 00.05]. Because the quadratic regression accounts for more variance in Enjoyment Ratings compared with the linear model (note the higher *R*^2^), our results indicate that the quadratic model better describes the relationship between *Average Information Content* and Enjoyment Ratings.

**Figure 5:**
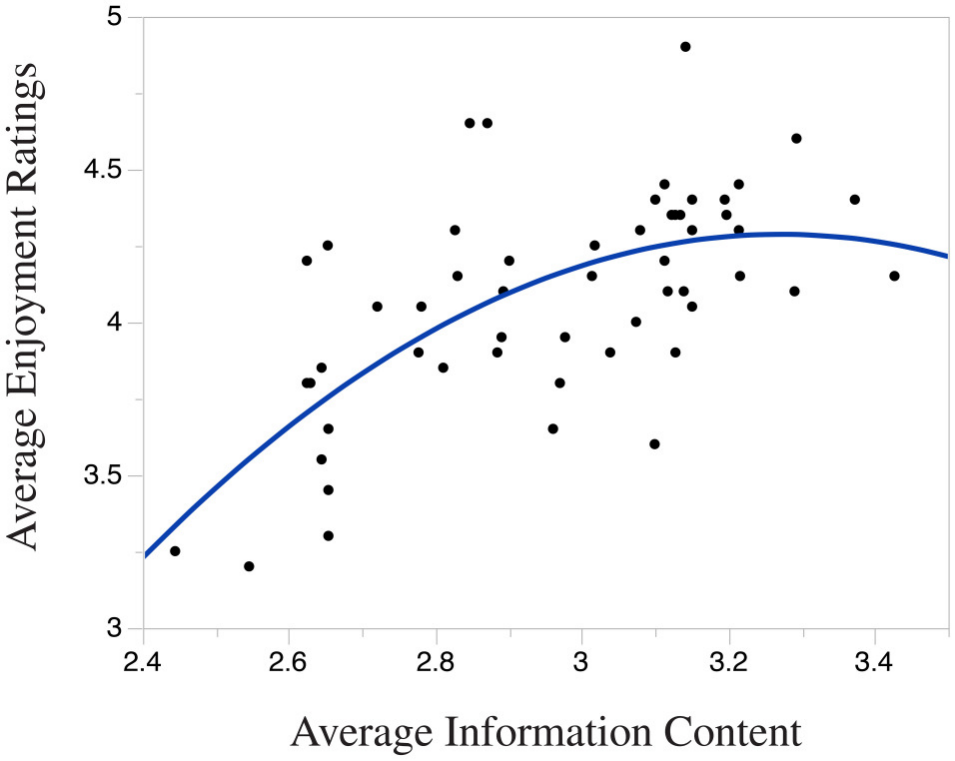
**Quadratic relation between IC and enjoyment**.

Figure 5 displays the quadratic regression function that maps Average Information Content to Average Enjoyment Rating. The graph shows the statistically significant inverse-U relationship between *Average Information Content* and enjoyment, which supports our second hypothesis.

### 3.2. Spiral array tension model

Increasing tension perceived through music is described by Farbood (2012) as “a feeling of rising intensity or impending climax, while decreasing tension can be described as a feeling of relaxation or resolution.” Because the focus of this research is primarily on the structure of chord transitions, we employ a computational model to assess the effect of tonal tension caused by chord changes.

**Figure 6:**
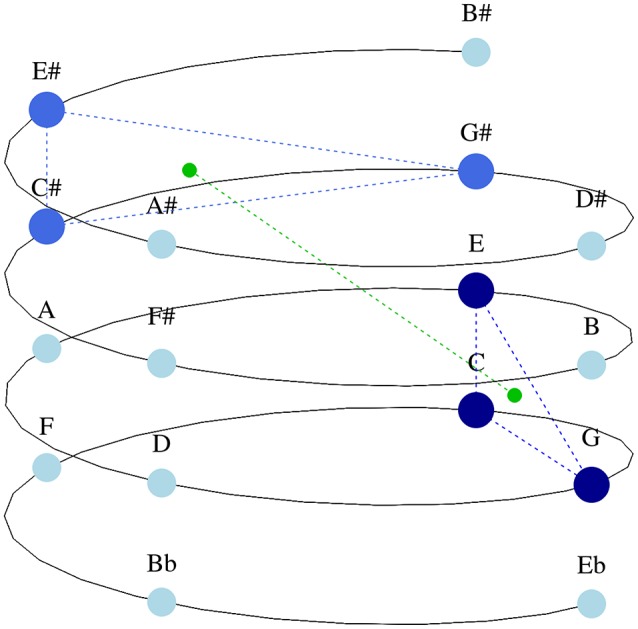
**C major (dark blue) and C♯ major (royal blue) triads together with their ***ce*** (small green dots) in the spiral array**. Other pitch classes are represented in light blue.

Herremans and Chew (2016a) developed a model that captures tonal tension based on the spiral array, a three-dimensional model for tonality (Chew, 2013). The spiral array is formed by three helices, which represent pitch classes, chords, and keys. The helix that embeds pitch classes is displayed in Figure 6. The spacial arrangement of the array causes close tonal relationships to be reflected by geometrical proximity, e.g., pitch classes next to each other form a perfect fifth, and those above each other a major third. The geometrical representation of pitches, chords and keys in the spiral array is inspired by earlier work on pitch spaces (such as that of Shepard, 1962; Krumhansl, 1979; Cohn, 1997). Chew (2013)'s model has been used for many applications, including key detection (Chuan and Chew, 2005), tonal segmentation (Chew, 2005), similarity assessment (Mardirossian and Chew, 2006), music generation (Herremans and Chew, 2016b), and pitch spelling (Chew and Chen, 2005).

When applying the model of Herremans and Chew (2016a), each chord of the UT anthem is represented as a cloud of points (each point in the array represents a note in the chord, and the points of a chord form a cloud) in the spiral array. Based on these clouds, three characteristics that capture different aspects of tonal tension may be calculated: Cloud Diameter, Tensile Strain, and Cloud Momentum. Cloud Diameter captures the largest tonal distance within a cloud (i.e., a chord). Because we only use major and minor triads in this study, this measure does not vary between the stimuli and has therefore been omitted. Tensile strain captures the tonal distance between a cloud and the global key. During stimulus generation in the present study, chords belonging to the global key of the anthem were chosen whenever possible. The impact of Tensile Strain on enjoyment is therefore minimal, and consequently not explored in this study. The present work focuses on the *Cloud Momentum* characteristic, which characterizes movement between chords in tonal space. In order to capture *Cloud Momentum*, we look at the “center of effect,” or *ce*, of each cloud, a feature which condenses the musical information of a cloud. The *ce* is the geometrical center of a cloud in the spiral array. By measuring the distance between the *ce*s of two subsequent clouds, we capture the movement (a proxy for perceived tension) in tonal space. A large shift between *ce*s reflects a change in tonality between two chords. In Figure 6, the movement between a C major and a C♯ major triad is represented in the array. The *ce* of both chords are marked as green points in tonal space. Because C and C♯ major chords are not tonally proximal, the distance between their center of effects in the helix is quite large (as displayed via the green line connecting the two points). In the current study, a triad representation of each chord was used in order to calculate the average *Cloud Momentum* for every sequence.

#### 3.2.1. Cloud momentum results

As for *Average Information Content*, the results for *Cloud Momentum* are provided via a comparison of linear and quadratic regression analyses, corresponding to hypotheses 1 and 2.

The linear regression indicates that *Cloud Momentum* explains a significant proportion of the variance in Average Enjoyment Ratings [$R^{2}=0.27,\;F_{\left(1,\;54\right)}=20.12,\;p<0.001$]. In this analysis, *Cloud Momentum* significantly predicts Enjoyment Ratings [β = 0.52, *t*_(54)_ = 4.49, *p* < 0.001], however, as with *Information Content*, the linear *R*^2^ is lower than that of the quadratic regression reported below.

The results of the quadratic regression indicate that *Cloud Momentum* explains a significant proportion of the variance in Average Enjoyment Ratings [$R^{2}=0.38,F_{\left(2,\;53\right)}=16.03,p<0.001$], where *Cloud Momentum* significantly predicts Enjoyment Ratings [β = 0.47, *t*_(53)_ = 4.28, *p* < 0.001], as does (*Cloud Momentum*)^2^ [β = −0.32, *t*_(53)_ = −3.00, *p* < 0.01]. Because this regression analysis accounts for more variability in Enjoyment Ratings (according to the linear and quadratic *R*^2^ values), the findings indicate that *Cloud Momentum* has an inverted-U relationship with Enjoyment.

**Figure 7:**
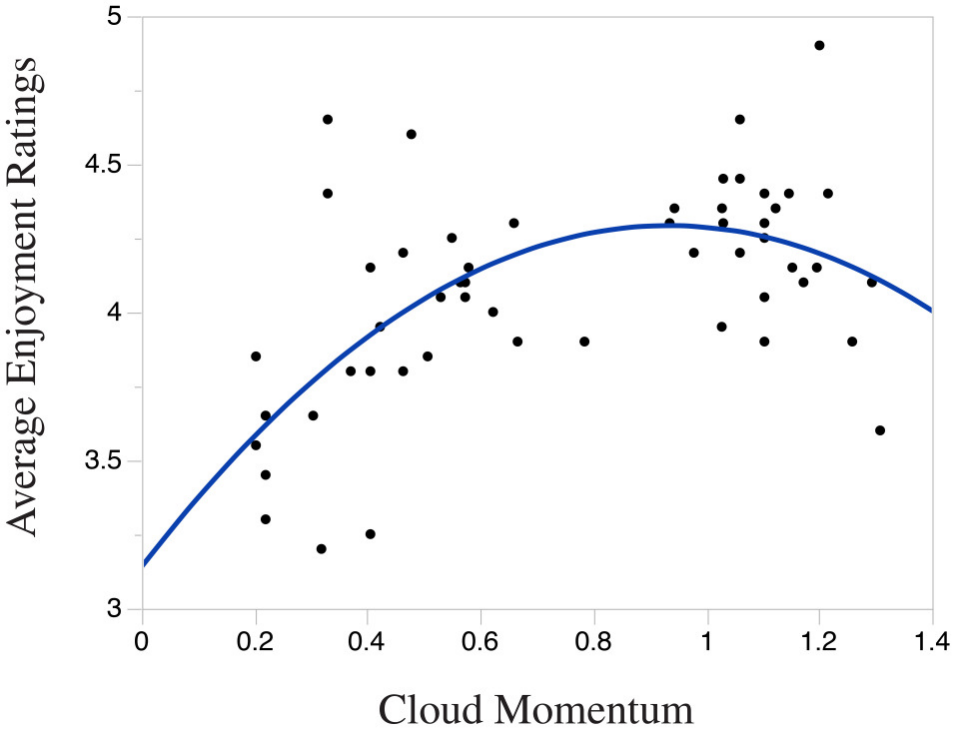
**Quadratic relationship between ***Cloud Momentum*** and enjoyment ratings**.

Figure 7 displays the significant inverse-U relationship between *Cloud Momentum* and Average Enjoyment Rating which again lends support for the Wundt curve hypothesis described above.

### 3.3. Discussion

Both measures of complexity modeled above yield a significant quadratic regression, and account for more variance in the data than their corresponding linear regression. Therefore, the results from modeling *Information Content* and *Cloud Momentum* provide support for the second hypothesis, that Enjoyment has an inverted-U relationship with repetitiveness/complexity (for the present research, more repetitive sequences are considered to be less complex). In both cases, increasing complexity leads to greater enjoyment, up to a certain point (the apex of the Wundt curve), after which enjoyment ratings begin to decrease.

## 4. General discussion

Taken together, the empirical and computational results indicate that the complexity of the underlying harmonic structure does have a measurable influence on the enjoyment of Uplifting Trance music. Overall the results provide greater support for the inverted-U relationship between complexity and enjoyment (hypothesis 2) than a direct linear relationship (hypothesis 1). Interestingly, however, there was an exception to this finding: Very repetitive stimuli (AABB-AABB) also elicit very high enjoyment ratings. An overview of the mechanisms driving enjoyment are discussed in detail below.

By systematically investigating the repetitiveness of semiotic structures, we have discovered specific contexts in which chord repetitions influence the enjoyment of UT music. At a general level, we found varied evidence that the semiotic structure of the first half of the stimulus sets up preferences for the remainder of the stimulus. For example, although listeners indicated greater enjoyment overall of anthems beginning with ABCD rather than AABB (that is, greater preference for more complex patterns), when stimuli began with the more *repetitive* semiotic structure of AABB, listeners preferred exact repetition more than chord variety. This may indicate that the initial harmonic structure sets up expectations and preferences for the later part of the semiotic structure. In other words, repetitive chords establish the expectation (and indeed, preference for) even more repetition of the semiotic pattern, whereas a more complex initial set of chords creates weaker expectations, allowing for a greater variety of harmonic instantiations to generate enjoyment. This result may be of interest to both trance music DJs and researchers in the area of computational creativity and music generation.

We also found that listeners prefer harmonic repetition within stylistically standard semiotic structures compared with structures that violate the implied form. For example, although the overall second-highest rated stimuli in the study were of the semiotic structure AABB-AABB, the lowest rated stimuli were of the structure AABB-BBAA, which contains the same chords, but the order of which violates expected harmonic progression. We believe that this connection between violation of form and dislike also contributes to the finding that listeners preferred stimuli with less chord repetition in the second half of the stimulus: The overall dislike of AABB-BBAA (as well as, to some extent, AABB-CCAA and AABB-AACC, where it could be argued that AA is not as expected as non-A chords) pulls down the average rating for structures with “2 unique chords in the second half.” Further, the second-lowest pattern, AABB-AAAA, violates the expectation for a change at the 7th chord position (and note that this is the only semiotic pattern which contains only “1 unique chord in the second half.” Further research is needed to fully tease apart how affective responses are linked to expected form and repetitiveness. In general, however, more evidence was found for the hypothesis 2, that a moderate level of complexity is desired.

Computational modeling of two measures of sequence complexity, *Average Information Content* and *Cloud Momentum* (average tonal tension of the sequence), provided support for an inverted-U relationship between complexity of harmonic structure and enjoyment. The full right-hand tail of the Wundt curve was not discovered because, as noted in the Section 2.1.4.2, only low information content sequences were included (sequences with high Information Content are low probability with respect to the statistical model used in generation, and were therefore non-stylistic, and often non-tonal, which warranted their exclusion from the present study). Nevertheless, the results indicate that increasing complexity generally yields higher Average Enjoyment Ratings, but that the most complex sequences (e.g., ABCD-EFGH) receive more modest ratings than the sequences with only moderately-complex structure (e.g., ABCD-BCDB).

The important message here, in the authors estimation, is that enjoyment of uplifting trance music, as measured via this experimental procedure, generally follows the Wundt curve, with the caveat that exact repetitions of semiotic structures (such as AABB-AABB) are also highly enjoyed. Familiarity and stylistic expectations of form also contribute to liking. We posit that experiences such as enjoyment (and resultant states of audience “flow”) in trance music may reflect a dynamic interplay between repetition and complexity.

Future work will explore the connection between repetitive harmonic elements and altered listening states signifying heightened enjoyment, such as audience “flow” and a heightened sense of group cohesion. To investigate this connection, future work will aim to contextualize this line of research within an appropriate setting; while the present study employs a controlled experimental design, which is necessary for systematically testing elements of trance harmonic structure, it does not reflect ecological listening conditions. Performing longer excerpts (with multiple loops of semiotic patterns) in a music venue, for example, would allow for greater ecological validity, and enable the exploration of resulting group listening behaviors. Presenting longer excerpts would also offer a closer approximation to the full trance listening experience (which is full of repetitive loops), and would therefore provide listeners with a more robust, phenomenological experience of Uplifting Trance music.

## Ethics statement

Approval for this research study was granted by the Queen Mary Ethics of Research Committee. Participants read an overview of the study, and received written and verbal instructions, prior to signing a consent form that indicated their informed consent to participate in the listening task. No vulnerable populations were included in this study.

## Author contributions

KA led the research project, although all authors were included in nearly every step of the research process. Specifically, KA led the theoretical motivation, experimental design, data collection, statistical analyses, interpretation of results, and writing. DH assisted with the experimental design, software for data collection, statistics, computational modeling, and writing. LB put together the corpus used for this study, rendered all stimuli, and wrote the material corresponding to these components in the Method section. DC was involved in the theoretical motivation, experimental design decisions, statistical analyses, statistical modeling, chord sequence generation, and he wrote the material describing the statistical model used for chord sequence generation.

### Conflict of interest statement

The authors declare that the research was conducted in the absence of any commercial or financial relationships that could be construed as a potential conflict of interest.

## Footnotes

^1^The Uplifting Trance Anthem Loops Corpus (UTALC), constructed specifically for this study, may be found here: http://www.lacl.fr/ lbigo/utalc

^2^http://www.logic-templates.com/product/uplifting-trance-logic-pro-x-template-by-insight

^3^The experiment software created for this research is available under an open source license at: http://dorienherremans.com/listener-experiment-software

^4^The Logic Pro file is available at: http://www.logic-templates.com/product/uplifting-trance-logic-pro-x-template-by-insight

^5^Please note that stimuli beginning AABB could only have 1 to 3 unique chords in the second half of the stimulus, and that stimuli beginning ABCD could only have 2 to 4 unique chords in the second half of the stimulus.

^6^Software that calculates the tonal tension based on the model of Herremans and Chew (2016a) is available online at http://dorienherremans.com/tension.
