# Supplementary material for: Harmonic Structure Predicts the Enjoyment of Uplifting Trance Music
Source: Front Psychol. 2017 Jan 10;7:1999. doi: 10.3389/fpsyg.2016.01999 (PMC5222838; doi:10.3389/fpsyg.2016.01999)
Supplement: Supplementary file 1 [file DataSheet1.pdf]

APPENDIX A: UPLIFTING TRANCE PIECES TRANSCRIBED IN THE CORPUS

Abstract Vision, MainGain - Freedom (Original Mix)
Adam Ellis - Velocity In French (Original Mix)
Alexandre Bergheau & Geert Huinink - Ephemere (Craft Integrated Remix)

Alexandre Bergheau - Call Me Up (Original Mix)
Allen & Envy and James Williams - Ark (Original Mix)
Allen & Envy feat Jess Morgan - The Heart That Never Sleeps (Allen Watts Remix)
Allen Watts - Kepler (Original Mix)
Allen Watts - Lifelines (Original Mix)
Aly & Fila & SkyPatrol feat Sue McLaren - Running (Original Mix)
Aly & Fila feat Ever Burn - Is It Love (Original Mix)
Aly & Fila with Jaren - For All Time (Extended Mix)
Andain - Beautiful Things (Fady & Mina Remix)
Andy Bianchini - Eastern Mountain (Original Mix)
ARDI - Addicted (Original Mix)
ARDI - Eternity (Original Mix)
ARDI - Kobia (Original Mix)
Armin van Buuren - Save My Night (Allen Watts Remix)
Ataraxia - Another Reason (Ula Remix)
Audrey Gallagher, Solis & Sean Truby - Skin Deep (Standerwick Remix)
Binary Finary & Lele Troniq feat Christina Novelli - Waiting For The Sun (Adam Ellis Remix)
Blue Horizon & Shyprince - Lithium (Original Trance Mix)
BluSkay & KeyPlayer - Cassiopeia (A & Z Remix)
BluSkay & KeyPlayer - Giza (Original Mix)
Bryan Kearney - Te Amo (Original Mix)
Chris Metcalfe & Allen Watts - Breakthrough (Original Mix)
Ciro Visone - First Coming (Ian Standerwick Remix)
Ciro Visone - Flying On The Moon (New World Remix)
Ciro Visone - Spring Dream (Original Mix)
Cold Rush - Pacific (Original Mix)
Daniel Kandi - Trancefamily (Original Mix)
Daniel Kandi - Child (Original Mix)
Darren Porter - Terraforming (Original Mix)
Davey Asprey - Anima (Original Mix)
Dimension & Ultimate - Corazon (Original Mix)
Dreamy - Legendary Dreamer (Original Energetic Mix)
Dreamy feat Isa Bell - Because You Are (Paul Rigel Remix)
Driftmoon - Effervescence (Dan Stone Remix)
Eddie Bitar & Christina Novelli - Start Again (Original Mix)
Elias B - Polar Ice (Adam Nickey Remix)
Etasonic Vs Laucco - Someone Like You (Emanuele Congeddu's Epic Take)
Farid - Afloat (Original Mix)

Gal Abutbul & Tim Briggs - Revert (Original Mix)
Gal Abutbul - Serenity (Original Mix)
Gareth Emery feat Bo Bruce - U (Bryan Kearney Remix)
Hydro Aquatic - Crossfire (Original Mix)
Ikerya Project - Lovely November (Original Mix)
Infinity & Ezietto - Distant Moon (Original Mix)
John Askew - Shine (Original Mix)
John Newall - Santa Monica (Original Mix)
John O'Callaghan feat Audrey Gallager - Big Sky (Adam Ellis Remix)
Jorn van Deynhoven - New Horzions (A state Of Trance 650 Anthem)
Laker - Breathing (Original Mix)
Las Salinas & Sue McLaren - Break The Spell (Bryan Kearney Remix)
Lee Osborne & Allen Watts - Telepathy (Original Mix)
Light & Wave - Feeling The City (Sunset Remix)
Matt Bukovski & Geert Huinink - Fields Of Forever (Original Mix)
Menno De Jong, Aneym - Your Heaven (Original Mix)
Moonsouls & Sunlab - Once In A Lifetime (Original Mix)
Nab Brothers - Diamond (Original Mix)
New World & Receptive - Whirling Dervishes (Original Mix)
New World - Fields Of La Tourette (Epic Mix)
New World - Ushio (Original Mix)
Onova - Platitude (Allen Watts Remix)
Overseas - Lot Of Nothing (The Noble Six Remix)
Paul Rigel & OBM Notion - Reminiscence (Original Mix)
Pedro Del Mar & Beatsole - Pianophoria (Original Mix)
Philippe El Sisi, Sarah Lynn - Look Above (Walsh & McAuley Remix)
Ram - RAMplify (Original Mix)
Ram feat Susana - RAMelia (Tribute To Amelia)
Re:Locate vs Robert Nickson & Carol Lee - Built To Last (Original Mix)
Rene Dale - Angel (Azima Remix)
ReOrder & Sue McLaren - Hands of Time (Dan Stone Remix)
ReOrder - Arrakis (Original Mix)
Seneta - Glasgow (Original Mix)
Sergey Nevone & Simon O'Shine - Apprehension (Aly & Fila Mix)
Sergey Nevone - White Swans (tranzLift Emotional Remix)
Sergey Shabanov - Illuminate (Gelardi Remix)
Solis & Sean Truby - The Climb (Solis And Sean Truby's Electronic Audio Outro)
Solis - True To Me (Suncatcher Remix)
SoundLift - Flying Higher (Original Mix)
South Pole - Oasis (Original mix)
Standerwick & Allen Watts - Next Generation (Original Mix)
Stevy Forello - Shaded Starlight (Temple One Remix)
Suncatcher - Are We There Yet?
Super8 & Tab - Irufushi (Original Mix)

Talla 2Xlc Feat Skye - Rise (Photographer Dub Remix)
Temple One - Unbreakable (Original Mix)
Temple One - Forever Searching (Adam Nickey Remix)
The Noble Six - Tapestry (Original Mix)
The Noble Six vs Chris Metcalfe - Ocean Avenue (Original Mix)
The Thrillseekers - When All Else Fails (Original Mix)
Touchstone & Tangle - Azure (Original Mix)
Tranzlift & Andy Elliass - Gates Of Albion (Paul Rigel Remix)

UCast - Klia (Original Mix)
UCast - LAX (Original Mix)
UCast - Tanity (Original Mix)
Ultimate - If We Were (Original Mix)
Will Atkinson - Victims (Original Mix)
Witness45 & Poonyk - Indiana (New World Remix)
Xgenic - Prometeus (Sensitize Remix)
